# Can anonymisation become disempowering? Rethinking ethics for low-risk global health research

**DOI:** 10.7189/jogh.15.03048

**Published:** 2025-11-14

**Authors:** Shukanto Das, Felicity Vidya Mehendale, Liz Grant

**Affiliations:** Usher Institute, Centre for Global Health, University of Edinburgh, Edinburgh, UK

## Abstract

Anonymisation is intended to confer protection in research, yet in research where disclosure poses minimal threat, blanket anonymisation may disempower participants who explicitly seek recognition. Drawing on fieldwork examining task shifting and sharing in India, we present an example where the leadership of a non-profit questioned why they must stay anonymous, viewing research participation as a rare chance to share learnings, document impact, and build credibility; opportunities otherwise constrained by funding and capacity limitations. Looking through an epistemic injustice lens, we argue that mandatory anonymisation policies reflect global north institutional assumptions about protection rather than the preferences of participants, potentially perpetuating patterns where researchers advance their goals whilst organisations whose knowledge forms their evidence remain invisible. While anonymisation is critical for sensitive research where disclosure could do harm, we call for flexible ethical guidelines that empower participants to make informed choices regarding recognition if risks are minimal. We recommend tiered consent processes which allow participants to select their levels of identification, differentiate between organisational and individual visibility, and use context-dependent frameworks like the Global Ethical Research toolkit to move towards proportionate, context-dependent decisions that truly protect those we study.

Fieldwork is key to global health studies within most postgraduate programmes and cross-border projects. For many researchers in high-income countries (HICs), this means travelling to low- and middle-income countries (LMICs) to collect data from frontline workers, organisations, or communities. Such engagements are often guided by ethics committees at institutions of the Global North, which may not necessarily align with participant expectations and realities [1]. In this viewpoint, we explore whether anonymity, while intended as protection [2,3], may in certain ways become disempowering. We then examine how anonymisation policies may reflect assumptions about participant protection over preferences they express. This discussion is grounded in an epistemic injustice framework [4], which explains how power shapes who is heard and whose knowledge is valued. What is generalisable from the case we present is who truly benefits from blanket anonymisation in research contexts where risk is minimal and recognition is wanted.

A PhD project at the UK’s Usher Institute is examining how task shifting and sharing (TS/S) are operationalised in India, building an implementation framework around best practices [5]. TS/S reallocate responsibilities among existing providers to tackle workforce shortfalls and have been used across domains [6], including filling mental health care gaps in LMICs [7]. Non-profits in underserved parts of India use TS/S, capitalising on community ties. The aforementioned project involved interviewing leadership, healthcare professionals, and community workers of such non-profits. Ethical approval for this investigation was granted by the Edinburgh Medical School Research Ethics Committee (EMREC), which required anonymising individuals and organisations recruited. Throughout data collection, participants across organisations expressed surprise about this requirement, suggesting a broader pattern of this phenomenon. Although anonymisation is meant to protect identity [2,3], we observed that it did not always suit participant interests. An exchange from a group interview with trustees of a non-profit in Gujarat that shifts screening, family counselling, and medication adherence support work for common mental health disorders [8] onto trained lay volunteers illustrates this tension (**Box 1**).

Box 1 **Transcript showing participant requests for recognition**

Participant A: You’ll not mention the name of (our) organisation?

Interviewer: No, the entire study is anonymous...

Participant B: Is it to remain anonymous?

Participant A: But in your dissertation or report, you’ll have to write, right?

Interviewer: We have to report it anonymously. We have details of the organisation in the (consent) form. So, for records, we know who we talked to, from which organisation... but it’ll be reported in a manner that this (finding) is (from) a non-profit based out of a city in Gujarat, which does such-and-such work. It’ll be anonymously reported. Even names of people I’m talking to, so, for example, your name, or people I interact with in the field, their names, won’t be reported to protect identities.

Participant A: No, no. We were thinking of sharing (your) report. It would be great to give names... so if anybody reads... your dissertation and wants to know more about (us)... they can contact you. What I think at the end of the report, or in references, the names of such organisations you are visiting should be there.

Participant B: Maybe so-and-so organisations have helped you gather this information?

Interviewer: So, unfortunately, as part of the protocol, I cannot, you know, write out the organisation name explicitly (in my thesis).

Participant B: No, at least in the thesis, it should be mentioned. That is a concern.

Participant A: Right.

To understand this, we must discuss what constitutes research risk. Risk is generally considered minimal when the probability and severity of harms are no greater than those experienced in everyday life [9,10]. It may be valuable to consider whether certain research may have no associated risks; this remains an under-explored notion, as there is often an assumption that even outwardly benign activities carry elements of uncertainty. Institutional ethics committees, including EMREC, prioritise anonymity to create safe spaces for participants, especially in high-risk scenarios where disclosure could harm [11,12]. This includes studies on sensitive topics where participants can face sociolegal or physical repercussions, like research on domestic violence, where revealing identities may endanger interviewees [13], or drug utilisation behaviour studies, where confidentiality breaches could lead to legal consequences [14], making anonymisation non-negotiable. In such studies, researchers adopt different strategies to safeguard identities, including using letters, numbers, and pseudonyms [15].

The UK’s Policy Framework for Health and Social Care Research [2] advises that regulation be proportional to risks participants are exposed to, and foreseeable benefits be weighed against said risks. The nature of our inquiry into TS/S was not sensitive, and no risks to interviewees (or indeed, the interviewer) were identified. Most cues in the topic guide [16] probed organisational management protocols and enablers and barriers to their TS/S. In low-risk scenarios such as ours, where participants actively sought recognition, blanket anonymisation created problems. As the transcript shows, organisations wanted to be visible, discuss their model, and connect with others doing similar work. Being named in published reports builds credibility that under-resourced small and medium non-profits doing innovative work in challenging settings value. For them, participating in HIC-endorsed research is a rare chance to share learnings broadly and document their local and regional impact – opportunities otherwise constrained by funding, prohibitive article processing charges [17], and lack of bandwidth to publish independently. Thus, being unnamed in reports is a missed opportunity for organisations to get visibility and hinders potential collaboration.

We note that the ask for recognition may itself be shaped by dependency relationships, pressures, or hopes for future partnerships, common in North-South collaborations [18]. Recognition may not necessarily mean empowerment either, as these inequalities can further prevent LMIC organisations from expanding independent research capacity. It is crucial, thus, to employ decolonising methodologies while engaging with such partners [19,20]. Even accounting for these power asymmetries, the flat denial of recognition when requested is a form of disempowerment that strips organisational agency in determining how they are credited. We recognise that our case offers one specific snapshot and that preferences vary. However, the question is not whether all universally desire recognition, but whether ones who explicitly ask for it should have that option if risks are minimal.

Bhakuni and Abimbola’s twin categories of testimonial and interpretative injustice [4] help understand why anonymisation can be problematic. Their epistemic injustice framework is driven by ‘pose’ (positionality from which knowledge is generated) and ‘gaze’ (the recipient of produced knowledge). These concepts show how the values associated with ‘participant’ and participants’ own sensemaking as they contribute to narratives are already limited by external systems that separately determine the nature of protection: who needs protecting, and its rationale; often without input from those being ‘protected.’ This way, in low-risk contexts, anonymisation may itself constitute injustice, wherein the power to determine participant benefit rests with institutional structures rather than the participants. When participants request recognition, their preferences come secondary to researchers’ or ethics committees’ assessments of what protection is. This imbalance is worrisome. Some have also raised anecdotally that anonymity may enable cover-ups of fabricated qualitative data [21]. While we lack evidence to examine these claims, they point to fundamental questions about power and accountability in global health research [22,23]: whose voices are trusted, whose work can be verified, and who benefits from opacity. This also creates a pattern where postgraduate students from HIC institutions study the works of organisations in LMICs, advancing their careers, while the people whose innovation or knowledge forms their evidence base stay invisible, despite disruption to their routine work or earning potential – an approach which has been critiqued as helicopter research [24]. Of course, not all HIC researchers are extractive, and not all LMIC partners desire recognition. We also understand that ethics committees themselves are constrained by legal frameworks such as GDPR, national data protection laws, and institutional liability concerns. Increasing litigation anxiety is driving greater conservatism in policies [25,26]. This cannot be ignored, and any reforms must be negotiated within existing legal boundaries. However, it is notable that outside academia, in business, industry, or civil society, there is more flexibility in acknowledging those with whom engagement has happened, suggesting that academic conservatism may exceed legal requirements.

Considering all this, we call for flexible ethical guidelines that empower participants to make informed choices regarding recognition if risks are minimal. This requires moving from blanket anonymisation to context-dependent policies. The Global Ethical Research toolkit [27], developed *via* consultation with over 200 researchers from over 30 countries, underscores that ‘ethics is contextual’ and robust processes must assess the ‘place’ (what works in this context), ‘people’ (what participants need), ‘principles’ (which values should guide us), and ‘precedent’ (what set practices need challenging) [28]. Applied to anonymisation, this requires examining the political climate, nature of the subject, power, participant vulnerabilities [29,30], and participant preferences. Also useful is to differentiate organisational and individual visibility. While organisations seek acknowledgement, individual staff may still want privacy. This nuance – that different anonymisation levels are needed for people within the same project – is often not accommodated by blanket policies. When risks are found to be negligible, ethics committees could consider tiered consent to allow participants to choose their levels of identification. This could offer, for example, total anonymity for those vulnerable; organisational acknowledgement with individual anonymity where institutions request recognition while staff ask confidentiality; or full attribution for those explicitly asking recognition in established low-risk contexts. Consent forms could explain the implications of these to make participants aware of their rights. This will also promote co-creation of research [31], *i.e.* research *with* participants rather than *on* them. However, this will need sincere efforts to get genuine consent, especially amidst power, knowledge, or poverty-related vulnerabilities. Participants may change preferences over time, requiring ways to revisit decisions. Documentation becomes complex, and researchers and ethics committees would need training to see signs of coercion and manage situations where different participants choose differently [18,32]. Ethics frameworks could also include opt-in acknowledgements of organisations in appendices, as suggested by our participants, or, at a minimum, let people select their own pseudonyms [33].

The conversation presented above makes one ponder what we are protecting people from and why. When organisations ask for recognition in low-risk contexts, mandatory anonymisation feels less like protection and more like paternalistic gatekeeping. Instead of forcing universal anonymity, ethics committees should differentiate between studies on sensitive topics needing stricter confidentiality measures and those where identity disclosure poses minimal threat and visibility offers benefits. What is at stake is not only individual preferences but a question of research justice. Since current anonymisation policies may silence the very voices we intend to safeguard, we must ask whether contemporary frameworks truly protect those we study or primarily protect institutions that study them.
